# Using personality as a predictor of diet induced weight loss and weight management

**DOI:** 10.1186/1479-5868-8-129

**Published:** 2011-11-23

**Authors:** Irene A Munro, Miles R Bore, Don Munro, Manohar L Garg

**Affiliations:** 1 Nutraceuticals Research Group, School of Biomedical Sciences & Pharmacy, The University of Newcastle, Callaghan NSW 2308, Australia; 2 School of Psychology. The University of Newcastle, Callaghan NSW 2308, Australia

## Abstract

**Background:**

A major challenge for successful weight management is tailoring weight loss programs to individual needs. The aim of this study was to investigate whether personality traits could be used to match individuals to a compatible weight loss program that would maximize weight loss.

**Method:**

Two different weight loss trials were conducted, both with a weight loss greater than 5% the measure of success. Fifty-four individuals, BMI 30-40 kg/m^2^, either followed a slow, healthy eating weight loss diet (HEWLD) of 5000-6000 kJ/day for 12 weeks (n = 22), or a fast, very low energy diet (VLED) of 3000 kJ/day for 4 weeks (n = 32). Anthropometric measurements were recorded at baseline, at the end of the weight loss period and, for VLED, at the end of 10 weeks of weight maintenance. Personality traits were measured at baseline using the Tangney Self Control Scale plus 3 of the scales from the Five Factor Model - Neuroticism, Conscientiousness and Extraversion.

**Results:**

The percentage weight loss was significantly greater in VLED (-7.38%) compared to HEWLD (-4.11%), (p < 0.001). Weight loss in HEWLD was positively correlated with Anxiety, a facet of Neuroticism. Weight loss in VLED was positively correlated with Neuroticism (r = 0.5, p < 0.01), and negatively correlated with Dutifulness and Discipline, facets of Conscientiousness, (p < 0.05 for both). No link was observed between weight loss and the personality trait, Self Control, in either HEWLD or VLED.

**Conclusion:**

The personality factor, Neuroticism, was linked to successful weight loss (that is ≥ 5%) with a particular weight loss treatment, suggesting that there is a potential to use measures of personality to identify appropriate weight loss/management strategies for individuals.

**Trial registration:**

Australia and New Zealand Clinical Trials Register (ANZCTR): ACTRN12611000716965

## Background

Obesity is a risk factor for several chronic diseases that are largely preventable, such as insulin resistance, type 2 diabetes mellitus, hypertension, dyslipidemia [1] and cardiovascular disease [2]. In addition to the significant morbidity and mortality that arises from these diseases, there is considerable social stigmatization associated with obesity. It has been suggested that a weight loss of 5-10% can significantly reduce the health risks [3,4] and there is no shortage of strategies available to assist with weight loss. Popular weight loss programs focus on weight reduction by restricting energy intake, either by reducing kilojoules (e.g. Weight Watchers), restricting fat intake (e.g. the Ornish diet), or restricting carbohydrate and increasing protein intake (e.g. the Atkins diet) [5]. Another strategy is a very low energy diet (VLED) using commercial meal replacements [6]. However, weight loss can be difficult to achieve or maintain and long term dietary compliance rates, are low, overall [7,8]. Restricting the intake of food in the face of hunger and temptation requires strong self control. Carels (2003) found that unsatisfactory weight loss and attrition during weight loss programs is associated with diminished self control [9]. When dietary control fails, other weight loss strategies may be sought including appetite suppressants such as Sibutramine, fat blockers such as Xenical or Orlistat [10], laxatives and diuretics, but these are not sustainable in the long term. More effective strategies to improve compliance rates for weight loss and weight maintenance are needed [5].

A major challenge for successful weight management is tailoring programs to meet individual needs, that is, matching personal attributes and behaviors to a particular weight loss program, such as whether a person with a particular personality will achieve a better weight loss outcome while following a time-convenient web-based weight reducing dietary program, for example, Collins 2010 [11], or in sessions that provide face-to-face personal support, for example, Cognitive Behavior Therapy [12]. Personality traits are measurable attributes of people and can be used to explain behavior. It is possible, therefore, that the profiling of personality traits could be matched with the various weight loss programs to identify those that could result in improved weight loss outcomes for the individuals concerned. Indeed, a number of studies have investigated the link between personality traits and weight loss with varying degrees of success [13-16]. However, their use of different tests to measure different traits makes it difficult to compare the findings.

Since about 1990, the Five Factor Model (FFM) [17] has become dominant in the study of personality, with a large proportion of studies of personality explicitly using measures based on it, or referring to it, as the standard approach. The 5 variables it proposes and their descriptive characteristics are Neuroticism (low self esteem, anxious, irritable and worrying), Conscientiousness (efficient, thorough, organized and hard working), Extraversion (socially stimulated, energetic, enthusiastic and pleasure seeking), Openness (imaginative, adventurous and spontaneous), and Agreeableness (sympathetic, gentle, trusting and warm) [18].

Associations between some of these personality traits and body weight and/or BMI have been reported. In different studies, Neuroticism has been found to be both positively and negatively correlated with obesity [18-20]. Conscientiousness is consistently associated with adiposity [19], with high Conscientiousness related to low BMI [18-20]. It has been suggested that there is less evidence for the association between BMI and the 3 remaining traits [19]; however, in different studies, high Extraversion has been correlated both positively and negatively with obesity [18,19]. Thus, there is evidence that 3 traits, Neuroticism, Conscientiousness and Extraversion, could provide a basis to identify personality attributes for successful weight loss. Another more specific trait likely to be predictive is Self Control. The Tangney Self Control Scale (SCS) [21] has been used in a number of studies to measure self control in relation to eating behaviors and weight [22,23] and was therefore included in the study.

The purpose of the present study was to determine whether the personality traits described above could be used to match individuals to a compatible weight loss program to maximize weight loss. For example, would a particular personality type respond better to a slow weight loss program using self-control to manage dietary compliance with a range of food choices, while another personality type might respond better to a more restrictive but faster weight loss? This was investigated with 2 groups of people using 2 different weight loss programs. We hypothesized that there would be a significant positive correlation between the personality trait, Self Control, and weight loss on a program that required participants to follow a healthy eating weight loss diet (HEWLD) and restrict their food intake without the aid of satiety enhancing supplementation. We were, however, uncertain which personality trait(s) would correlate with weight loss on a program that removed food choices and reduced feelings of hunger with satiety enhancing meal replacements. Therefore we explored the relationship between weight loss and the 3 FFM dimensions that have been found to correlate with weight control, Conscientiousness, Neuroticism and Extraversion, and the 6 facets for each (as listed Table 1).

**Table 1:** Correlations of personality with percentage weight loss and BMI loss for HEWLD and VLED, and weight maintenance for VLED

	HEWLD		VLED		
	Weight loss	BMI change	Weight loss	BMI change	Maintenance
	r (n = 22)	r (n = 22)	r (n = 32)	r (n = 32)	r (n = 29)
**Self Control**	-.293	-.291	-.302	-.290	-.209
**Neuroticism:**	.274	.272	.500**	.503**	.494**
Anxiety	.406	.411	.411*	.394*	.381*
Depression	.135	.128	.436*	.495**	.472**
Self consciousness	.143	.128	.563**	.553**	.422*
Vulnerability	.081	.089	.375*	.360*	.424*
Anger	.258	.269	.480**	.485**	.472**
Immoderation	.300	.288	.167	.151	.220
**Conscientiousness:**	-.106	-.096	-.299	-.286	-.169
Dutifulness	-.066	-.049	-.376*	-.345	-.150
Self-discipline	-.226	-.212	-.364*	-.377*	-.231
Self-efficacy	-.014	-.013	-.291	-.294	-.252
Cautiousness	-.180	-.188	-.075	.002	.064
Orderliness	-.019	-.005	-.142	-.156	.019
Achievement-striving	.042	.051	-.026	-.046	-.168
**Extraversion**	.021	.036	-.236	-.355	.106
Friendliness	-.088	-.074	-.174	-.258	-.113
Gregariousness	-.164	-.176	-.245	-.320	-.088
Assertiveness	.037	.049.	-.294	-.378	.318
Excitement-seeking	.113	.126	-.068	-.213	.152
Cheerfulness	.083	.080	-.133	-.221	.136
Activity level	.175	.182	-.074	-.125	.178

*p < 0.05, **p < 0.01 (2-tailed)

## Methods

### Participants

Both male and female participants were recruited from the university campus and the general community in Newcastle, Australia, to take part in one of two different weight loss/management trials. For both trials participants were required to have a BMI of between 30 - 40 kg/m^2 ^and be aged 18 - 60 years. People with diagnosed diabetes mellitus, a chronic inflammatory condition, or who were already following an energy restricted diet, were excluded from the study, as were women who were pregnant or lactating. This study was conducted in accordance with the guidelines laid down in the Declaration of Helsinki and approved by the Human Research Ethics Committee of the University of Newcastle, Australia. Written, informed consent was obtained from participants prior to commencement. The trial was registered with the Australian New Zealand Clinical Trials Registry (ACTRN12611000716965)

### Study design

Two weight loss/management trials were conducted with participants having no prior knowledge that the 2 different trials were to be offered. Initially participants were recruited to take part in a weight loss study that provided for a slow and steady loss of weight over 12 weeks. Later, following the same process, participants were recruited to take part in the second study that provided for a quick weight loss over a short period of time, 4 weeks, and was based on meal replacements. A weight loss greater than 5% was considered as the measure of success. To ensure uniformity of information presented all information given to the participants was provided by one person, and included nutrition education and counseling sessions which were conducted over the first 4 weeks of the trials. The nutrition education sessions focused on the energy density of foods, understanding and using food labels, appropriate portion sizes as well as the number of portions to be consumed daily from the different food groups. This information was used to help participants build a healthy diet using the guidelines from the Australian Guide to Healthy Eating (AGHE) [24].

In the first trial participants followed a portion controlled, reduced energy, HEWLD comprising 5000 kJ for females to 6000 kJ for males daily for 12 weeks. The diet was based on the AGHE [24] which allowed participants to eat a variety of healthy foods, albeit a reduced amount. The changed eating patterns, enabling a steady weight loss, are considered to be a sound strategy for the development of life-long healthy eating habits. However, if the stomach is accustomed to holding and digesting larger volumes, managing hunger could be a problem and dietary compliance difficult. With the second trial participants followed a very low energy diet (VLED) of 3000 kJ/day for 4 weeks. For the first 2 weeks participants received Optifast^® ^bars and shakes to replace meals according to the Intensive Phase of the Optifast^® ^Very Low Calorie Diet Program (Novartis, Australia) [25], supplemented daily with raw and cooked vegetables and 2 liters of drinking water. This is a rigid diet and the shakes and bars are satiety enhancing because of the high polydextrose content and/or high protein content, which helps to reduce hunger and facilitate compliance. The lack of variety of food choice is very restricting but enables a faster weight loss over a shorter period. During weeks 3 and 4, the meal replacements were gradually phased out and healthy kilojoule controlled meals were phased in so that participants would learn to choose healthy foods and correct portion sizes, based on the AGHE. Immediately following the 4 weeks of weight loss, and still observing the principles of the AGHE, participants progressed to 10 weeks of weight maintenance to reinforce healthy eating behaviors. This meant that the weight loss/management period was then similar for both groups. An accredited practicing dietitian was part of the research team to advise on health care.

### Instruments

Prior to commencement, individual diets were assessed with a 3-day food diary to determine every-day nutrient and energy intake. The diaries were analyzed using the program FoodWorks Professional 2009, version 6 (Xyris Software (Australia) Pty Ltd.) and the mean values calculated.

At the same time, 2 questionnaires were given to participants to measure personality. There are several self-report instruments based on the FFM available; in this study, a public-domain test based on the work of L.R. Goldberg (2006) was used [26], namely The International Personality Item Pool http://ipip.ori.org 'Big Five' questionnaire. This instrument has been shown to be highly reliable in many investigations using it and has been used locally for several years so local norms are available [27]. Three of the 5 scales were used, Neuroticism, Extraversion and Conscientiousness but not the other 2 as previous research has not found them to be clearly related to dietary weight loss behavior. There were 10 items for each of 6 'facets' (subscales), to a total of 180 items, each rated on a 5-point scale. Both facets and scale totals were used to test specific relationships with weight gain/loss behaviors. The FFM was used in conjunction with a second instrument, the Tangney Self Control Scale (SCS) which measures general self-control, such as the ability to refrain from acting on undesired behavioral tendencies [21]. This 36-item scale has been shown to be reliable (alpha coefficient = .85) and has been used previously in research on eating styles [22,28].

On the first day of the weight loss trial, anthropometric measurements were taken in the morning after ≥ 10 hour overnight fast, with participants dressed in light clothing and without shoes. Standing height was measured to the nearest 0.1 cm using a stadiometer. Body weight was measured to the nearest 0.1 kg using a calibrated balance beam scale (PCS Measurement, NSW, Australia). BMI was calculated in kilograms per meter squared from weight and height. Under identical conditions, these anthropometric measurements were repeated at the end of the weight loss phase as well as at the end of the maintenance phase in the second trial, the VLED.

### Statistical analyses

ANOVA was used to test group mean differences, and Pearson product-normal correlations (r) were used to show relationships, with p ≤ 0.05 indicating statistical significance (critical value for r = 0.423 for n = 22, and r = 0.349 for n = 32, 2-tailed). Multiple regression analysis was used to test the possibility that weight loss was a function of the interaction of group (treatment) with personality.

## Results

Twenty two participants (8 males) completed the HEWLD and 32 participants (6 males) completed the VLED trials. There was no significant difference in age, baseline body weight, BMI and daily energy intake between the 2 groups (Table 2).

**Table 2:** Descriptive characteristics of participants at baseline

	HEWLD (n = 22)		VLED (n = 32)	
	
	Mean	SD	Mean	SD
Age (years)	41.0	3.0	42.0	2.0
Body weight (kg)	94.2	14.7	91.2	13.7
BMI (kg/m^2^)	32.3	2.6	32.9	3.2
Daily kJ intake	7875.0	2897.0	8086.0	1991.0

(Mean values and standard deviations)

**Figure 1:**
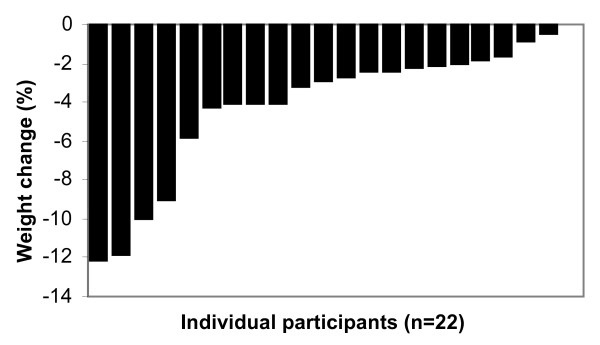
**Individual weight change during the 12 week weight loss phase of HEWLD**.

**Figure 2:**
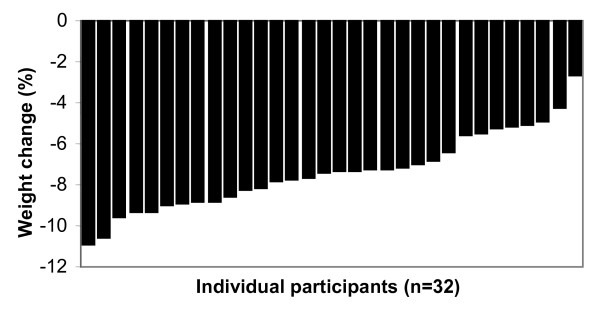
**Individual weight change during the 4 week weight loss phase of VLED**.

A weight loss of at least 5% has been suggested as necessary to significantly reduce the health risks arising from obesity [3,4]. Only 5 of the 22 participants following the HEWLD had lost 5% or more of their body weight after 12 weeks with a mean weight loss and BMI reduction of 4% for both. The changes from baseline were not significantly different. After 4 weeks of the VLED, the mean body weight of participants had reduced by 7% (p = 0.02) and BMI had also reduced by 7% (p = 0.002). All but 2 of these participants had achieved a weight loss of 5% or above. The weight loss between the 2 groups was significantly different (p < 0.001); the individual differences in weight loss for both groups shown in Figures 1 and 2.

**Figure 3:**
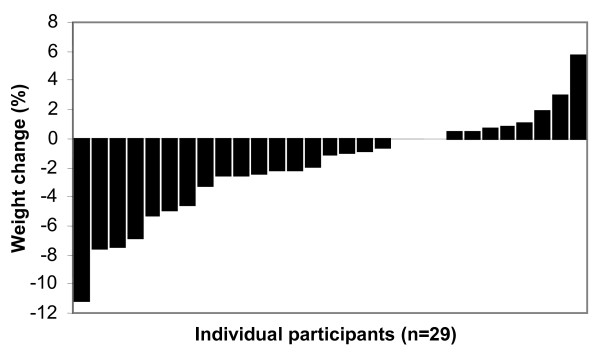
**Individual weight change during the 10 week weight maintenance phase of VLED**.

Three participants chose not to continue to the maintenance phase of the VLED. Twenty-nine participants completed the 10 weeks of weight maintenance, 2 of whom regained 2-3% (1.7-2.5 kg) and one person regained just under 6% weight (4.3 kg). Three participants did not change their weight at all, 6 lost a further 4-7% and one lost a further 11% weight. The others continued to lose or gain small amounts (Figure 3).

The mean personality scores for HEWLD and VLED are shown in Table 3; there were no significant differences between the 2 groups. In the current obese sample, scale reliabilities as measured by Cronbach's alpha for the main personality traits were .86 for Neuroticism, .79 for Extraversion, .80 for Conscientiousness, and .85 for Self Control, all of which are satisfactory. The correlations between personality (both facet and whole scale scores) and percentage weight loss and percentage BMI change for both HEWLD and VLED, and between personality and percentage weight maintenance for VLED, were computed (Table 1). For the HEWLD there were no significant correlations between personality and weight loss or BMI change. However, for the VLED group, there was a significant, positive correlation between the Neuroticism personality trait and weight loss, BMI change and Maintenance change, in particular the facets Anxiety, Anger, Depression, Self consciousness and Vulnerability, with significance ranging from < 0.05 to 0.001, but not the facet, Immoderation. Weight loss and BMI change were also negatively correlated with the Conscientiousness facet, Discipline, and weight loss with the facet, Dutifulness, (p < 0.05 for all). None of the facets of personality within the factor Conscientiousness were significantly correlated with weight maintenance. For both HEWLD and VLED there were no significant correlations between Extraversion (both facet and whole scale scores) and weight loss or BMI change, or between Self Control in the Tangney SCS and weight loss or BMI change. It should be noted that though the correlation between change in the weight loss phase and the maintenance phase for the VLED group was r = 0.48, indicating moderate independence between the phases, the patterns of correlations within personality were similar. Because of the small sample sizes it was not practicable to apply a Bonferroni or similar correction to compensate for the large number of comparisons, so the results should be interpreted with caution.

**Table 3:** Personality scores for participants at baseline

**Personality Traits**	**HEWLD (n = 22)**			**VLED (n = 32)**			
		
	**Mean**	**SD**	**Norm**	**Mean**	**SD**	**Norm**	**Alpha**
			z *			z*	
	
Neuroticism	134.6	23.9	-0.45	137.6	25.5	-0.33	.86
Extraversion	162.3	20.4	-0.32	165.0	20.0	-0.18	.79
Conscientiousness	181.2	18.6	+0.63	178.8	14.8	+0.50	.80
Self Control	118.5	13.8	+0.21	117.3	17.2	+0.15	.85

*With respect to undergraduate norms with n = 237 for IPIP scales, Tangney et al (2004) for Self Control

Multiple regression analysis was used to check that there were no significant interactions between groups (as treatments) and personality. None were found, partly due to the small sample sizes.

## Discussion

The aim of this study was to investigate the possibility that different personality traits are differentially related to different weight loss diets so that individuals might in future be matched to treatments to maximize weight loss. In particular, we hypothesized that there would be a significant positive correlation between the personality trait, Self Control, and weight loss on a program that required participants to follow a healthy diet and restrict their food intake without the aid of satiety enhancing supplementation (HEWLD). No specific predictions were made about which personality trait(s) would correlate most strongly with weight loss on the other program that initially removed food choices but reduced feelings of hunger with satiety enhancing meal replacements (VLED).

Using 2 different weight loss strategies, we found there was a significant difference in weight change from baseline in the VLED group, but the change in weight loss from baseline was not significantly different for the HEWLD group. A lack of willpower or self-discipline is often blamed for the inability to lose and manage weight but controlling hunger is not easy. Participants in the HEWLD were required to reduce their food intake and to eat healthy foods. This reduction in food intake would have been associated with increased feelings of hunger requiring considerable self control to resist the immediate temptation to assuage the pangs of hunger for the delayed reward of weight loss. The mean scores for self control were similar for HEWLD and the VLED, 118.5 and 117.3 respectively. For both trials, the correlations between self control and weight change or BMI change are negative and not significant. In particular, the treatment outcome in the HEWLD, that is low weight loss, was not associated with our Self Control measure. A study by Carels (2003) investigating failure to lose weight during treatment with 44 obese, postmenopausal women found that one of the significant influences on poor treatment outcome was diminished self control [9], but they attributed the poor treatment outcomes to a number of other causes as well. A study which examined the control of eating (restraint, measured using the TFEQ [29]) and a possible association with general self control (measured using the SCS [21]) found that, among the obese participants and dieters, restraint was related to more successful weight control [22]. They found that overweight and obese participants had higher restraint scores compared to those of normal weight; however, normal weight participants had higher self control scores. Also, current and past dieters had higher restraint scores compared to never-dieters; however never-dieters had higher self control scores. We did not measure eating restraint in our study, but the findings of the study by Konttinen (2009) appear to indicate that self control is not associated with the control of food intake among overweight and obese individuals and current and past dieters. It has been suggested that, overall, self control is unrelated to decision making [30]. The study found that, in their daily lives, individuals who were high in self control made less frequent references to positive and negative affect and physiological states, for example feeling tired and hungry, and were seen as being less spontaneous compared to individuals with low self control [30]. They suggest that self control may inhibit affective experiences in general. Tangney (2004) suggests that individuals that are high in self control favor long-term goals in the guidance of their behavior [21], so it is possible that these individuals are not concerned with the immediate issues of their weight and are focused on other aspects of their lives. While the HEWLD was underpowered to determine significant correlations, there was no association between weight loss and self control in the VLED group either.

It is possible that an alternative measure for self control is the trait Conscientiousness. In the current study Conscientiousness and Tangney Self Control were highly correlated (r = .769 for HEWLD and r = .701 for VLED, p < .001 for both). Previous research using the FFM has reported significant negative correlations between Conscientiousness and adiposity in males and females; that is, high Conscientiousness was associated with a low BMI [18-20]. In particular, the facets "order" and "self discipline" were strongly associated with weight [20]. Sutin (2011) suggests that people with high scores in these facets are thinner because they are organized and follow their diet and meal plans [19]. However, it should be noted that these studies were about personality profiles and weight/BMI, not about weight loss [18-20]. In the current study the correlations between Conscientiousness and weight loss were negative and modest for both groups apart from the 2 facets "dutifulness" and "self discipline" that were significantly and negatively correlated with weight loss in the VLED group. If, as Sutin suggests, those that are high in Conscientiousness are thinner, then there is less need for them to follow a weight loss regime. Thus, in the current sample, high Conscientiousness could be comparatively lower in this group wanting to lose weight, and that may also explain the conflicting results compared to previous studies that did not focus on weight loss. It is also possible that people high in Conscientious do not respond well to the weight loss regimes followed in this study.

Despite prior research reporting that high Extraversion was associated with a high BMI [19] and a high BMI in males but not in females [18] our study found no significant relationship between Extraversion and weight loss or BMI in this group.

The VLED participants consumed satiety enhancing meal replacements and, with hunger initially controlled, the small bars and shakes enabled the stomach to reduce in size so that when healthy meals were phased in to the diet, participants were satisfied with less food. The satiating effect of the Optifast^® ^bars and shakes appear to have facilitated dietary compliance in the VLED group, as evidenced by > 5% weight loss in all but 2 of the participants over 4 weeks. In this group there was a significant positive correlation between weight loss and Neuroticism and its facets "anxiety, anger, depression, self consciousness and vulnerability" but not "immoderation".

Previous studies on personality and adiposity have reported that high Neuroticism is significantly associated with a high BMI in females [18] and a high BMI in both males and females [19]. Elfhag (2005) suggests that disinhibited eating is related to the factor Neuroticism [31]. It has also been suggested that unrestrained eaters, who eat as a natural response to hunger and appetite, appear to reduce their food intake more radically with enhanced satiety, resulting in greater weight loss [32]. A study by Elfhag (2008), using the NEO Personality Inventory-Revised which is based on the FFM of personality [33], reported that weight loss supplemented with Sibutramine, which enhances satiety, was significantly and positively correlated with the factor Neuroticism, and 2 of its facets, 'anxiety' and 'depression' [34].

There is also the suggestion that Sibutramine appears to be more effective with people who eat as a natural response to hunger and not as a response to cognition and conscious control [34]. Optifast^® ^provides a similar satiating effect to Sibutramine and weight loss with both showing a significant correlation with Neuroticism. It is possible that the VLED group was also people who responded to hunger and appetite rather than control for weight loss but our study did not measure eating behavior so this cannot be confirmed.

The VLED continued to maintenance after the 4 weeks of weight loss to determine whether the participants could continue to manage their weight, and for this phase participants continued consuming a diet based on the AGHE. This extended their trial to match the time span of the HEWLD. Although weight loss for the 2 phases, that is weight loss and maintenance, was only moderately correlated, the correlations with personality are very similar.

Some studies have found no link between personality and weight loss. The Dutch Personality Questionnaire which includes 7 scales; Neuroticism, Social anxiety, Rigidity, Hostility, Egoism, Dominance and Self-esteem was used with gastric banding for weight loss and the findings showed that none of the personality variables was associated with weight outcomes [14]. The 10-item Life Orientation Test was used to determine whether dispositional optimism had a predictive value for weight loss with no significant results [35]. A study by Poston [15] used the Karolinska Scales of Personality to predict weight loss and found that the personality traits identified by the Karolinska Scales of Personality did not predict weight loss. We hypothesized that self control would be associated with being able to limit food intake and restrict food choices so it was interesting to observe that, in our study, there was no link between weight loss and self control in the Tangney SCS. However there do not appear to be any studies which positively associate the personality attribute self control with weight loss.

Schwartz (1995) surveyed a group of obesity experts to determine whether or not they agreed about factors important for matching individuals to weight loss treatment [36]. She refers to previous research that suggests that people with different personality styles, levels of obesity and disturbed eating practices will respond differently to the various treatments. The position of the American Dietetic Association on weight management is that it is important to find ways to optimize individualized treatments appropriately [37]. Using measures of personality traits to identify appropriate weight loss and weight management strategies remains an intriguing possibility, but so far none of the relationships that have been found are reliable enough to base treatment regimes on them.

## Conclusion

This was an exploratory study to determine whether there was a relationship between personality and different methods of weight loss. The results of this study have shown that there is a link between the personality factor, Neuroticism, and successful weight loss with a particular weight loss treatment that facilitates dietary compliance with enhanced satiety. These results were unexpected as Neuroticism normally has an inhibited effect on behavior [38]; those who are higher in Neuroticism are more likely to be anxious, depressed and have poor self esteem and so do not perform as well in many things they do. As noted above, Elfhag (2005) also found associations between neuroticism and eating behavior, but much remains to be clarified about these relationships. Surprisingly to us, there were no significant relationships with weight loss and Self-Control, as one might expect self control would play a part in behaviors related to weight control. It was predicted that the HEWLD would show such an association, as compliance with that weight loss diet required self control. Unfortunately the sample size for this group was too small to show significant results. Overall, the number of participants involved in this study was relatively small so the results should be interpreted with caution. It is also clear that both theory and measurement methods require further development for the links between personality and weight management behavior to be understood.

## Competing interests

The authors declare that they have no competing interests.

## Authors' contributions

All authors contributed to the concept and design of the research project. IM conducted the trials and collected the data, analyzed and interpreted the data, and drafted the manuscript. DM analyzed and interpreted the data and drafted components of the manuscript. MB, DM, and MG provided essential materials and provided critical review. All authors read and approved the final manuscript.

## References

[B1] Fruhbeck G Fantuzzi G, Mazzone T Vasoactive Factors and Inflammatory Mediators Produced in Adipose Tissue Adipose Tissue and Adipokines in Health and Disease 2007 Totowa: Human Press Inc 63 77

[B2] Poirier P Giles TD Bray GA Hong Y Stern JS Pi-Sunyer FX Eckel RH Obesity and cardiovascular disease: pathophysiology, evaluation, and effect of weight loss: an update of the 1997 American Heart Association Scientific Statement on Obesity and Heart Disease from the Obesity Committee of the Council on Nutrition, Physical Activity, and Metabolism Circulation 2006 113 898 918 10.1161/CIRCULATIONAHA.106.171016 16380542

[B3] Blackburn G Effect of degree of weight loss on health benefits Obes Res 1995 3 Suppl 2 211s 216s 8581779 10.1002/j.1550-8528.1995.tb00466.x

[B4] Goldstein DJ Beneficial health effects of modest weight loss Int J Obes Relat Metab Disord 1992 16 397 415 1322866

[B5] Dansinger ML Gleason JA Griffith JL Selker HP Schaefer EJ Comparison of the Atkins, Ornish, Weight Watchers, and Zone diets for weight loss and heart disease risk reduction: a randomized trial Jama 2005 293 43 53 10.1001/jama.293.1.43 15632335

[B6] Heymsfield SB van Mierlo CA van der Knaap HC Heo M Frier HI Weight management using a meal replacement strategy: meta and pooling analysis from six studies Int J Obes Relat Metab Disord 2003 27 537 549 10.1038/sj.ijo.0802258 12704397

[B7] Hill JO Thompson H Wyatt H Weight maintenance: what's missing? J Am Diet Assoc 2005 105 S63 66 15867898 10.1016/j.jada.2005.02.016

[B8] Van Buren DJ Sinton MM Psychological aspects of weight loss and weight maintenance J Am Diet Assoc 2009 109 1994 1996 10.1016/j.jada.2009.09.010 19942015

[B9] Carels RA Cacciapaglia HM Douglass OM Rydin S O'Brien WH The early identification of poor treatment outcome in a women's weight loss program Eat Behav 2003 4 265 282 10.1016/S1471-0153(03)00029-1 15000970

[B10] Halford JC Pharmacotherapy for obesity Appetite 2006 46 6 10 10.1016/j.appet.2005.07.010 16229924

[B11] Collins CE Morgan PJ Jones P Fletcher K Martin J Aguiar EJ Lucas A Neve M McElduff P Callister R Evaluation of a commercial web-based weight loss and weight loss maintenance program in overweight and obese adults: a randomized controlled trial BMC Public Health 2010 10 669 10.1186/1471-2458-10-669 21047432 PMC2989963

[B12] Cooper Z Doll HA Hawker DM Byrne S Bonner G Eeley E O'Connor ME Fairburn CG Testing a new cognitive behavioural treatment for obesity: A randomized controlled trial with three-year follow-up Behav Res Ther 2010 48 706 713 10.1016/j.brat.2010.03.008 20691328 PMC2923743

[B13] Elfhag K Rossner S Carlsson AM Barkeling B Sibutramine treatment in obesity: predictors of weight loss including rorschach personality data Obes Res 2003 11 1391 1399 10.1038/oby.2003.188 14627761

[B14] Larsen JK Geenen R Maas C de Wit P van Antwerpen T Brand N van Ramshorst B Personality as a predictor of weight loss maintenance after surgery for morbid obesity Obes Res 2004 12 1828 1834 10.1038/oby.2004.227 15601979

[B15] Poston WS Ericsson M Linder J Nilsson T Goodrick GK Foreyt JP Personality and the prediction of weight loss and relapse in the treatment of obesity Int J Eat Disord 1999 25 301 309 10.1002/(SICI)1098-108X(199904)25:3<301::AID-EAT8>3.0.CO;2-P 10191995

[B16] Sullivan S Cloninger CR Klein S Personality characteristics in obesity and relationship with successful weight loss International Journal of Obesity 2007 31 669 674 16953251 10.1038/sj.ijo.0803464 PMC4450078

[B17] McCrae RR Costa PT Jr Validation of the five-factor model of personality across instruments and observers J Pers Soc Psychol 1987 52 81 90 3820081 10.1037//0022-3514.52.1.81

[B18] Brummett BH Babyak MA Williams RB Barefoot JC Costa PT Siegler IC NEO personality domains and gender predict levels and trends in body mass index over 14 years during midlife Journal of Research in Personality 2006 40 222 236 10.1016/j.jrp.2004.12.002

[B19] Sutin AR Ferrucci L Zonderman AB Terracciano A Personality and obesity across the adult life span J Pers Soc Psychol 2011 101 579 592 21744974 10.1037/a0024286 PMC3462003

[B20] Terracciano A Sutin AR McCrae RR Deiana B Ferrucci L Schlessinger D Uda M Costa PT Jr Facets of personality linked to underweight and overweight Psychosom Med 2009 71 682 689 10.1097/PSY.0b013e3181a2925b 19414622 PMC2711222

[B21] Tangney JP Baumeister RF Boone AL High self-control predicts good adjustment, less pathology, better grades, and interpersonal success J Pers 2004 72 271 324 10.1111/j.0022-3506.2004.00263.x 15016066

[B22] Konttinen H Haukkala A Sarlio-Lahteenkorva S Silventoinen K Jousilahti P Eating styles, self-control and obesity indicators. The moderating role of obesity status and dieting history on restrained eating Appetite 2009 53 131 134 10.1016/j.appet.2009.05.001 19433123

[B23] Kuijer R de Ridder D Ouwehand C Houx B van den Bos R Dieting as a case of behavioural decision making. Does self-control matter? Appetite 2008 51 506 511 10.1016/j.appet.2008.03.014 18479777

[B24] Kellet E Smith AE Schmerlaib Y The Australian Guide to Healthy Eating 1998 Canberra: Australian Government Department of Health and Ageing

[B25] Optifast Very Low Calorie Diet http://www.optifast.com.au/why-optifast-vlcd.aspx

[B26] Goldberg LR Johnson JA Eber HW Hogan R Ashton MC Cloninger CR Gough HG The international personality item pool and the future of public-domain personality measures Journal of Research in Personality 2006 40 84 96 10.1016/j.jrp.2005.08.007

[B27] Munro D Bore MR Powis DA Personality factors in professional ethical behaviour: studies of empathy and narcissism Australian Journal of Psychology 2005 57 49 60 10.1080/00049530412331283453

[B28] Elfhag K Morey LC Personality traits and eating behavior in the obese: poor self-control in emotional and external eating but personality assets in restrained eating Eat Behav 2008 9 285 293 10.1016/j.eatbeh.2007.10.003 18549987

[B29] Karlsson J Persson LO Sjostrom L Sullivan M Psychometric properties and factor structure of the Three-Factor Eating Questionnaire (TFEQ) in obese men and women. Results from the Swedish Obese Subjects (SOS) study Int J Obes Relat Metab Disord 2000 24 1715 1725 10.1038/sj.ijo.0801442 11126230

[B30] Zabelina DL Robinson MD Anicha CL The psychological tradeoffs of self-control: A multi-method investigation Personality and Individual Differences 2007 43 463 473 10.1016/j.paid.2006.12.015

[B31] Elfhag K Personality correlates of obese eating behaviour: Swedish universities Scales of Personality and the Three Factor Eating Questionnaire Eat Weight Disord 2005 10 210 215 16755164 10.1007/BF03327487

[B32] Elfhag K Rossner S Barkeling B Rooth P Sibutramine treatment in obesity: initial eating behaviour in relation to weight loss results and changes in mood Pharmacol Res 2005 51 159 163 10.1016/j.phrs.2004.07.005 15629262

[B33] Costa PT Jr Fagan PJ Piedmont RL Ponticas Y Wise TN The five-factor model of personality and sexual functioning in outpatient men and women Psychiatr Med 1992 10 199 215 1615160

[B34] Elfhag K Finer N Rossner S Who will lose weight on sibutramine and orlistat? Psychological correlates for treatment success Diabetes Obes Metab 2008 10 498 505 10.1111/j.1463-1326.2007.00740.x 17593239

[B35] Fontaine KR Cheskin LJ Optimism and obesity treatment outcomes J Clin Psychol 1999 55 141 143 10.1002/(SICI)1097-4679(199901)55:1<141::AID-JCLP15>3.0.CO;2-G 10100841

[B36] Schwartz MB Brownell KD Matching individuals to weight loss treatments: a survey of obesity experts J Consult Clin Psychol 1995 63 149 153 7896981 10.1037//0022-006x.63.1.149

[B37] Seagle HM Strain GW Makris A Reeves RS Position of the American Dietetic Association: weight management J Am Diet Assoc 2009 109 330 346 19244669 10.1016/j.jada.2008.11.041

[B38] Eysenck HJ Pervin LA Biological dimensions of personality Handbook of personality: Theory and research 1999 New York: Guildford 244 276

